# Antibiotic action and resistance: updated review of mechanisms, spread, influencing factors, and alternative approaches for combating resistance

**DOI:** 10.3389/fphar.2023.1305294

**Published:** 2024-01-12

**Authors:** Esraa M. Halawa, Mohamed Fadel, Mohammed W. Al-Rabia, Ali Behairy, Nehal A. Nouh, Mohamed Abdo, Rada Olga, Liana Fericean, Ahmed M. Atwa, Mohammad El-Nablaway, Ahmed Abdeen

**Affiliations:** ^1^ Department of Botany and Microbiology, Faculty of Science, Cairo University, Giza, Egypt; ^2^ Department of Microbial Chemistry, Biotechnology Research Institute, National Research Centre, Dokki, Cairo, Egypt; ^3^ Department of Clinical Microbiology and Immunology, Faculty of Medicine, King Abdulaziz University, Jeddah, Saudi Arabia; ^4^ Department of Clinical Laboratories-Diagnostic Immunology Division, King Abdulaziz University Hospital, Jeddah, Saudi Arabia; ^5^ Department of Pharmacology, Faculty of Medicine, Benha University, Benha, Egypt; ^6^ Department of Microbiology, Medicine Program, Batterjee Medical College, Jeddah, Saudi Arabia; ^7^ Inpatient Pharmacy, Mansoura University Hospitals, Mansoura, Egypt; ^8^ Department of Animal Histology and Anatomy, School of Veterinary Medicine, Badr University in Cairo (BUC), Badr City, Egypt; ^9^ Department of Anatomy and Embryology, Faculty of Veterinary Medicine, University of Sadat City, Sadat City, Egypt; ^10^ Department of Biology and Plant Protection, Faculty of Agriculture, University of Life Sciences “King Michael I” from Timișoara, Timișoara, Romania; ^11^ Department of Pharmacology and Toxicology, Faculty of Pharmacy, Egyptian Russian University, Cairo, Egypt; ^12^ Department of Medical Biochemistry, Faculty of Medicine, Mansoura University, Mansoura, Egypt; ^13^ Department of Basic Medical Sciences, College of Medicine, AlMaarefa University, Riyadh, Saudi Arabia; ^14^ Department of Forensic Medicine and Toxicology, Faculty of Veterinary Medicine, Benha University, Toukh, Egypt

**Keywords:** antibiotics, antimicrobial, bacteria, antibiotic resistance, disease spreading

## Abstract

Antibiotics represent a frequently employed therapeutic modality for the management of bacterial infections across diverse domains, including human health, agriculture, livestock breeding, and fish farming. The efficacy of antibiotics relies on four distinct mechanisms of action, which are discussed in detail in this review, along with accompanying diagrammatic illustrations. Despite their effectiveness, antibiotic resistance has emerged as a significant challenge to treating bacterial infections. Bacteria have developed defense mechanisms against antibiotics, rendering them ineffective. This review delves into the specific mechanisms that bacteria have developed to resist antibiotics, with the help of diagrammatic illustrations. Antibiotic resistance can spread among bacteria through various routes, resulting in previously susceptible bacteria becoming antibiotic-resistant. Multiple factors contribute to the worsening crisis of antibiotic resistance, including human misuse of antibiotics. This review also emphasizes alternative solutions proposed to mitigate the exacerbation of antibiotic resistance.

## 1 Introduction

Microbiology originated the term “antibiotic” from the French words “antibiose” and “antibiotique,” as defined by Vuillemin in the late 19th century to delineate substances that exert detrimental effects on living organisms, particularly microorganisms (Laskin, Bennett, and Gadd, 2002). Subsequently, in 1947, Selman A. Waksman provided an all-encompassing definition of antibiotics as chemical compounds produced by microorganisms that possess the capacity to impede the growth and induce the demise of bacteria and other microorganisms (Waksman, 1947; Kaur Sodhi and Singh, 2022). Antibiotics have found widespread application across various sectors, encompassing human health, agriculture, livestock breeding, and fish farms (Okocha, Olatoye, and Adedeji, 2018; Kaur Sodhi and Singh, 2022). The mechanisms underlying the efficacy of antibiotics involve four distinct modes of action (Kaur Sodhi and Singh, 2022), including inhibition of DNA replication (Fàbrega et al., 2009), protein biosynthesis (Tenson, Lovmar, and Ehrenberg, 2003), cell wall biosynthesis (Cho, Uehara, and Bernhardt, 2014), and folic acid metabolism (Saverus 2019). However, the emergence of antibiotic resistance has escalated into a critical global issue (Sarkar et al., 2021; Shree et al., 2023).

This review article comprehensively discusses the multifaceted factors contributing to the propagation of antibiotic resistance, alongside providing potential strategies for mitigating this problem. Furthermore, innovative solutions to combat antibiotic resistance have been uncovered by scientists, such as the utilization of nanoantibiotics, and antibiotic adjuvants, the discovery of novel antibiotics, and the exploration of alternatives to antibiotics, including bacteriophages and botanicals. In addition to exploring the aforementioned topics, this review article delves into the utilization of antibiotics in diverse sectors, the controversies encircling their use, the mechanisms by which antibiotics act, the emergence of antibiotic resistance, the mechanisms governing antibiotic resistance in bacteria, the dissemination of resistance among bacterial populations, and the pivotal factors that contribute to the escalation of resistance. Moreover, this article highlights the accomplishments achieved thus far and presents solutions that scientists have uncovered to combat the global crisis of antibiotic resistance.

## 2 Antibiotics use

Antibiotics are utilized in a variety of sectors, including agriculture, aquaculture, animal husbandry, and human health (Okocha et al., 2018; Kaur Sodhi and Singh, 2022) (Table 1). These substances are used to treat bacterial infections in humans, animals, and crops, thereby preventing crop loss from bacterial diseases (Svircev, Roach, and Castle, 2018; Kaur Sodhi and Singh, 2022). Additionally, antibiotics are widely used as growth-promoting agents in animal husbandry (Sriram et al., 2021; Kaur Sodhi and Singh, 2022). The utilization of antibiotics in livestock is classified into three categories by scientists (Gonzalez Ronquillo and Angeles Hernandez, 2017): therapeutic agents, prophylactic agents, and growth promoters. Therapeutic agents are administered in high doses to infected animals to treat illnesses (Schwarz, Kehrenberg, and Walsh, 2001), while prophylactic agents are given in sub-therapeutic doses through feed or drinking water to prevent disease when there are no evident symptoms of infection. Antibiotics are periodically administered to animals throughout their life cycle (Greene and Pisano 2012). Growth promoters are used to improve an animal’s growth rate and production, and a tiny quantity of antibiotics is regularly administered through its feed (Wierup, 2001). In aquaculture, antimicrobial substances are used to treat fish infections. Fish are given antibiotics by incorporating them into specially formulated feed, and they mostly excrete them into the environment (Dawood, Koshio, and Esteban, 2018). It is worth noting that approximately 75% of the antibiotics provided to fish are excreted into the water (Benbrook, 2002).

**TABLE 1:** Antibiotic class use in different sectors.

Sector	Antibiotic use
Human health	- Treatment of bacterial infections.
Animal husbandry	- Treatment of bacterial infections.
	- Growth promoting agents.
Agricultural activities	- Prevention of crop loss from bacterial diseases.
Aquaculture	- Treatment of fish diseases.

## 3 Mechanisms of action of antibiotics

Not all antibiotics have the exact mechanism of action; thus, scientists classify antibiotics according to their mechanism of action and chemical structure (Kaur Sodhi and Singh, 2022) into four mechanisms described as follows:

### 3.1 Antibiotics inhibit DNA replication

**FIGURE 1:**
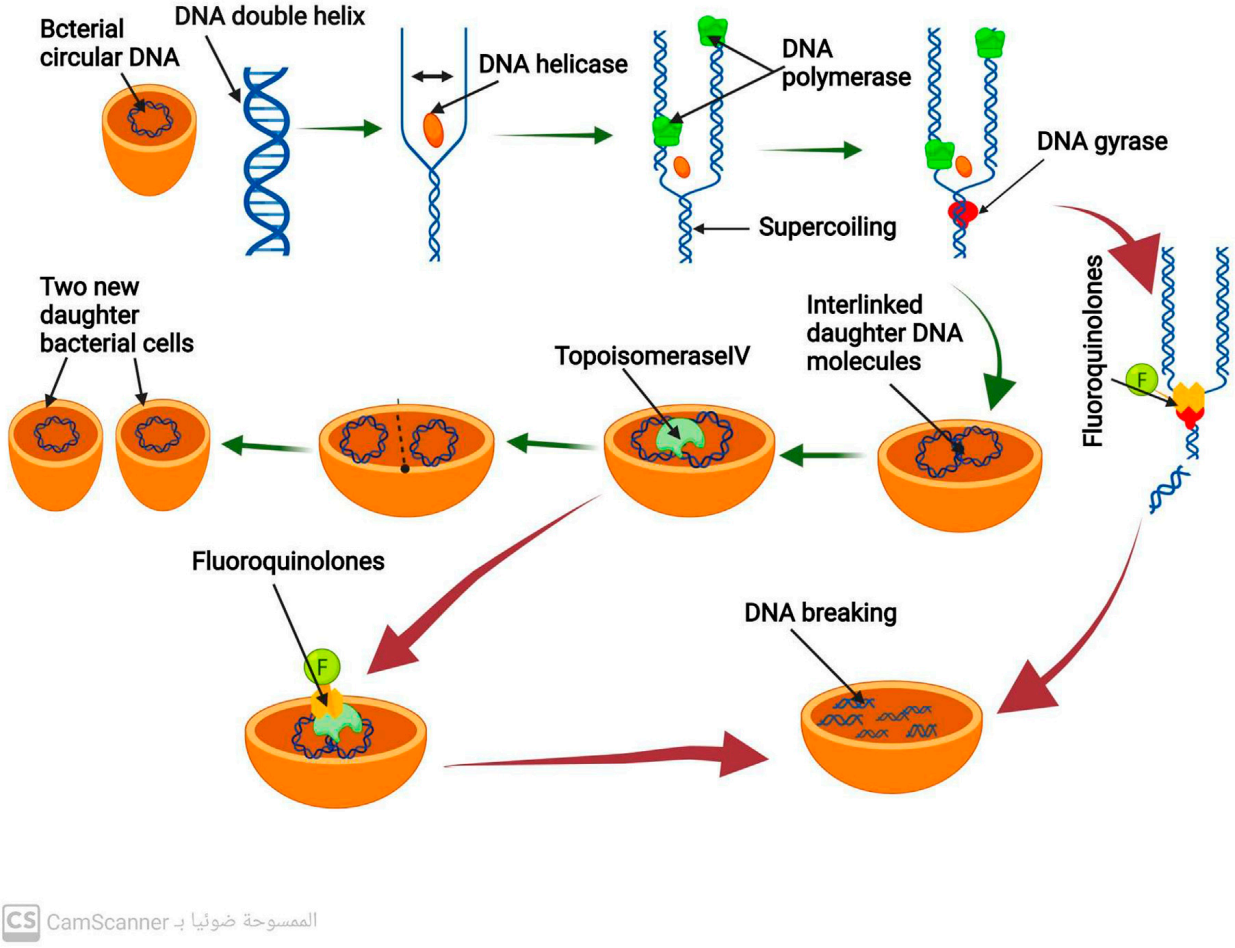
Mechanism of DNA replication process and mechanism of action of antibiotics that inhibit DNA replication.

Binary fission is a type of cell division used by bacteria that produces two daughter cells (Bhattacharyya, 2012). Before that can happen, however, bacteria must create exact duplicates of their circular DNA. DNA replication is the procedure used to duplicate DNA (Gilbert, 2001). A DNA double helix strand is split into two single strands by the enzyme DNA helicase to begin this process. The enzyme DNA polymerase then produces new DNA Strands that are complementary to the old ones (Li and Araki, 2013). Positive DNA helical twists build up as a result of DNA helicase and DNA polymerase activity. If not eliminated, these positive helical twists stop DNA replication from continuing. The removal of the positive superhelical twists is carried out by the enzyme DNA gyrase, also known as topoisomerase II, allowing DNA replication to continue (Bush, Evans-roberts, and Maxwell, 2015). A crucial bacterial enzyme called DNA gyrase is made up of 2A and 2B subunits. This enzyme also plays a crucial role in the transcription of several genes and the start of DNA replication. Once the new two daughter DNA molecules have been created, they eventually connect and are interlinked. In order to divide the bacterial cell into two new daughter bacterial cells, the enzyme topoisomerase IV (related to DNA gyrase) enables the separation of the two connected DNA molecules (Nagaraja et al., 2017) (Figure 1).

### 3.2 Fluoroquinolones antibiotics

By inhibiting the activity of DNA gyrase and topoisomerase IV, fluoroquinolone antibiotics prevent the synthesis of bacterial DNA (Fàbrega et al., 2009; Shree et al., 2023). These antibiotics have a particular affinity for binding to the complex formed by DNA gyrase and DNA (Pham, Ziora, and Blaskovich, 2019). Such binding destabilizes the enzyme-DNA complex, causing DNA cleavage and ultimately leading to bacterial cell death (Malik, Zhao, and Drlica, 2006) (Figure 1). Fluoroquinolones primarily target DNA gyrase, which is the reason for their effectiveness against most gram-negative bacteria (Blondeau, 2004).

In contrast, in most gram-positive bacteria, fluoroquinolones target topoisomerase IV as their primary mechanism (Blondeau, 2004). However, they also act as a secondary target for DNA gyrase. This results in the binding of fluoroquinolones to DNA and the complex formed by topoisomerase IV, which causes disruption of the separation of the two daughter DNA molecules and eventually leads to DNA breakage (Malik et al., 2006).

### 3.3 Antibiotics inhibit protein biosynthesis

Like all other living things, bacteria have DNA, which contains the genetic information for every protein they need to survive. This includes the protein needed for metabolism regulation, growth, repair, and reproduction (Wu, 2009). Additionally, it encodes for mRNA, rRNA, and tRNA, three types of RNA that are essential for carrying out protein synthesis (Wu, 2009).

**FIGURE 2:**
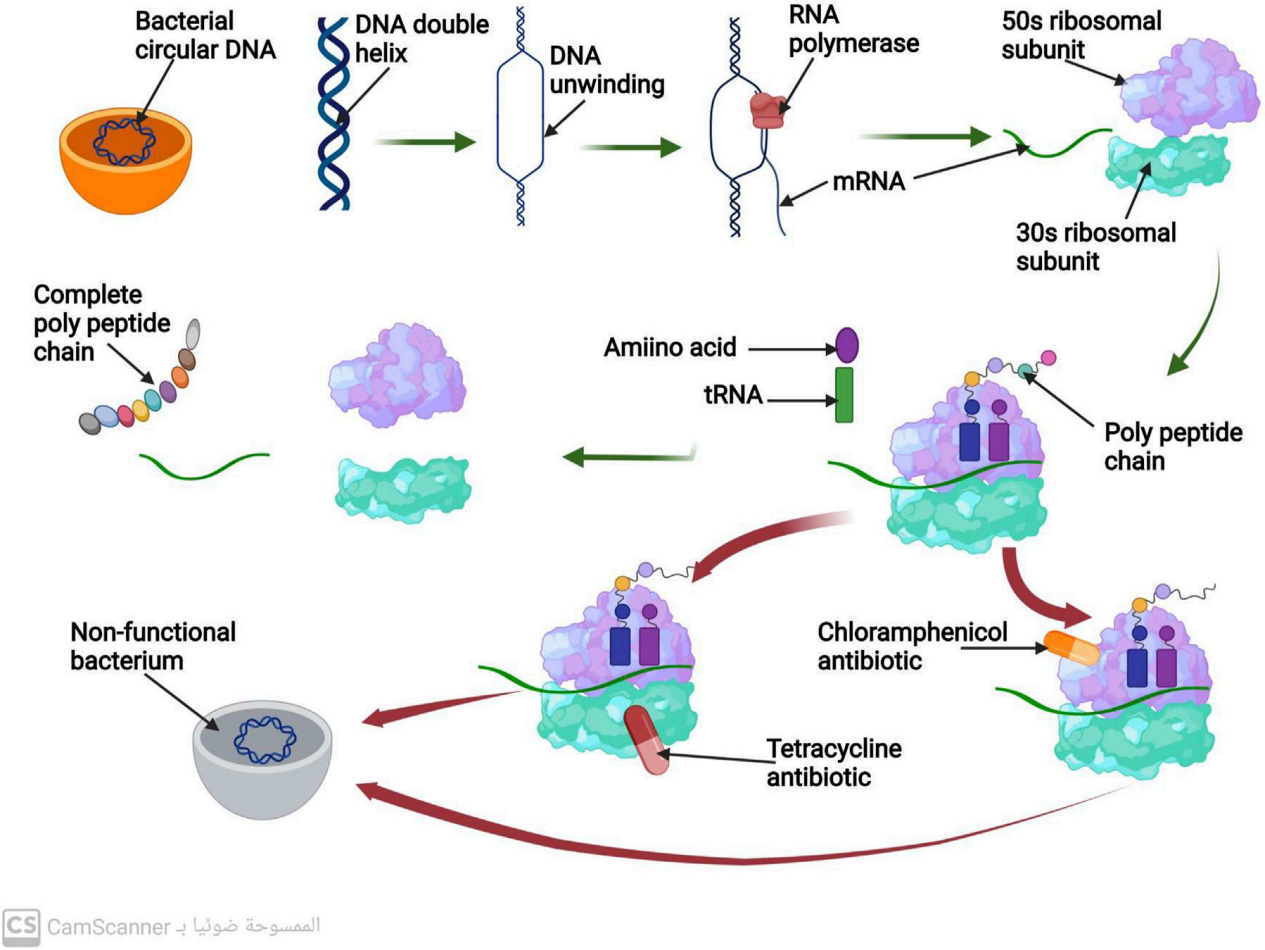
Mechanism of protein biosynthesis process and mechanism of action of antibiotics that inhibit protein biosynthesis.

The unwinding and separation of the DNA molecule at a region that codes for the necessary protein to be produced is the first step in the process of protein biosynthesis. The transcription, the process of making mRNA, uses only one strand of DNA as a scaffold. When the mRNA strand is finished, it separates from the DNA template and then is connected to a ribosome. The 50 s and 30 s ribosomal subunits make up the bacterial ribosome. Following the joining of these two subunits along the mRNA strand, the synthesis of the polypeptide chain starts. Up until it encounters the signal along the mRNA to stop, the ribosome keeps adding amino acids to the lengthening polypeptide chain. The full polypeptide chain is generated at this point (Johansson, Lovmar, and Ehrenberg, 2008) (Figure 2). Therefore, targeting the ribosomal 30 or 50 subunits is necessary for antibiotics to inhibit protein synthesis (Scott Champney, 2008).

#### 3.3.1 Antibiotics inhibit protein biosynthesis by targeting the ribosomal 305 subunits

##### 3.3.1.1 Aminoglycosides antibiotics

Antibiotics known as aminoglycosides function through a specific mechanism. These antibiotics are positively charged, which attracts the negatively charged outer membrane of bacteria, causing the membrane to develop large pores (Delcour, 2009). The aminoglycosides are then able to enter the bacterial cell through these pores. Additionally, aminoglycosides are able to pass through the bacterial cytoplasmic membrane by utilizing the energy of active bacterial transport (Jana and Deb, 2006). The target of aminoglycosides is the 16s rRNA of the 30s, which they bind to via hydrogen bonds. This binding inhibits protein biosynthesis before it can be completed (Vicens and Westhof, 2003).

Although aminoglycosides are effective against many types of bacteria, they have low efficacy against anaerobic bacteria, as these bacteria require oxygen for active transport pathways to function (Kohanski, Dwyer, and Collins, 2010). However, when combined with an antibiotic that inhibits cell wall synthesis, aminoglycosides have been found to have a greater ability to penetrate bacterial cells at low doses (Wang et al., 2022) (Figure 2).

##### 3.3.1.2 Tetracycline antibiotics

This class of antibiotics targets the highly conserved sequence of the 16S rRNA present in the ribosomal 30S subunit. Tetracycline, for instance, functions by hindering the binding of tRNA to the A-site of the ribosome, which ultimately impedes the process of protein synthesis (Brodersen et al., 2000).

#### 3.3.2 Antibiotics inhibit protein biosynthesis by targeting the ribosomal 50s subunit

##### 3.3.2.1 Macrolides antibiotics

Macrolide antibiotics bind to the 50S subunit of the ribosome, thereby preventing the synthesis of polypeptide chains and inhibiting protein production (Tenson et al., 2003) (Figure 2).

##### 3.3.2.2 Chloramphenicol antibiotics

Chloramphenicol antibiotics inhibit peptidyl transferase, an enzyme located on the 50S ribosomal subunit that is necessary for protein synthesis. This inhibition prevents t-RNA from connecting to the ribosomal A site, leading to the inhibition of protein synthesis (Syroegin et al., 2022) (Figure 2).

##### 3.3.2.3 Oxazolidinone antibiotics

Oxazolidinone antibiotics prevent the synthesis of the initiation complex by binding to the 50S subunit of the ribosome, thereby preventing the production of proteins (Foti et al., 2021) (Figure 2).

### 3.4 Antibiotics inhibit cell wall synthesis

**FIGURE 3:**
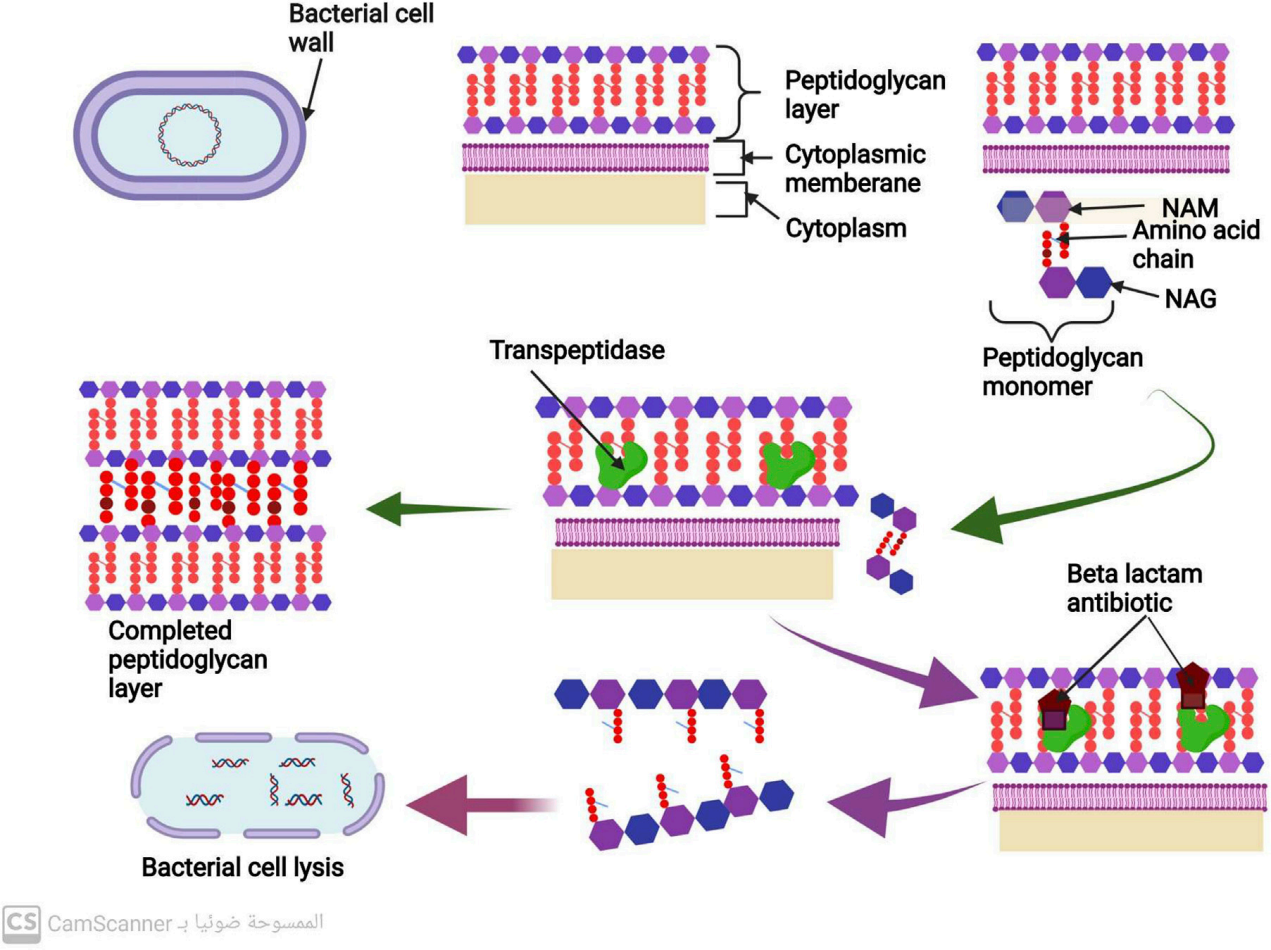
Mechanism of cell wall synthesis and Mechanism of action of antibiotics that inhibit cell wall synthesis.

Most bacteria are composed of a cell membrane enclosed in a cell wall, while some bacteria also have an extra outer layer. The bacterial cell wall serves two purposes: to keep the bacteria in their distinctive shape and to stop them from bursting when osmosis is used to introduce fluid into the cell (Gupta and Gupta, 2021). The peptidoglycan is the most significant part of the cell wall. N-acetyl muramic acid (NAM) alternates with N-acetyl Glucosamine (NAG), and the two are connected by chains of amino acids to form the polymer known as peptidoglycan (Meroueh et al., 2006). There are several steps in the peptidoglycan synthesis process, which ultimately results in bacterial cell wall formation. A precursor to peptidoglycan is created by combining N-acetyl glucosamine (NAG) with N-acetyl muramic acid. The cell wall acceptors in the periplasm receive this peptidoglycan precursor after it has been delivered across the membrane. The peptidoglycan precursors attach to cell wall receptors in the periplasm and go through a lot of cross-linking (Liu and Breukink, 2016). Trans peptidase and carboxy peptidase enzymes are the two main enzymes in cross-linking. A cell wall eventually develops from many peptidoglycan layers that are all cross-linked (Liu and Breukink, 2016). Gram-positive bacteria may have a thicker cell wall than gram-negative bacteria because they may contain more layers of peptidoglycan (Figure 3) (Pasquina-Lemonche et al., 2020).

The peptidoglycan is the most significant part of the bacterial cell wall. Transpeptidase and carboxypeptidase are the two primary enzymes in cross-linking during cell wall biosynthesis (Liu and Breukink, 2016) (Figure 3).

#### 3.4.1 Betalactams antibiotics

All penicillins and cephalosporins with the beta-lactam ring chemical structure are included in this group (Fernandes, Amador, and Prudêncio, 2013). This distinctive structure allows them to attach to peptidoglycan cross-linking enzymes, such as transpeptidase and carboxypeptidase, ultimately inhibiting bacterial cell wall synthesis and preventing cross-linking (Cho et al., 2014). This inhibition of cell wall manufacturing leads to the destruction of the bacterial cell, as shown in Figure 3 (Cho et al., 2014).

#### 3.4.2 Glycopeptides antibiotics

These antibiotics form non-covalent bonds with the terminal carbohydrates, which prevents the cross-linking of peptidoglycan precursors. This process ultimately leads to the degradation of bacterial cell walls, resulting in the destruction and elimination of bacterial cells (Kang and Park, 2015).

### 3.5 Antibiotics inhibit folic acid metabolism

**FIGURE 4:**
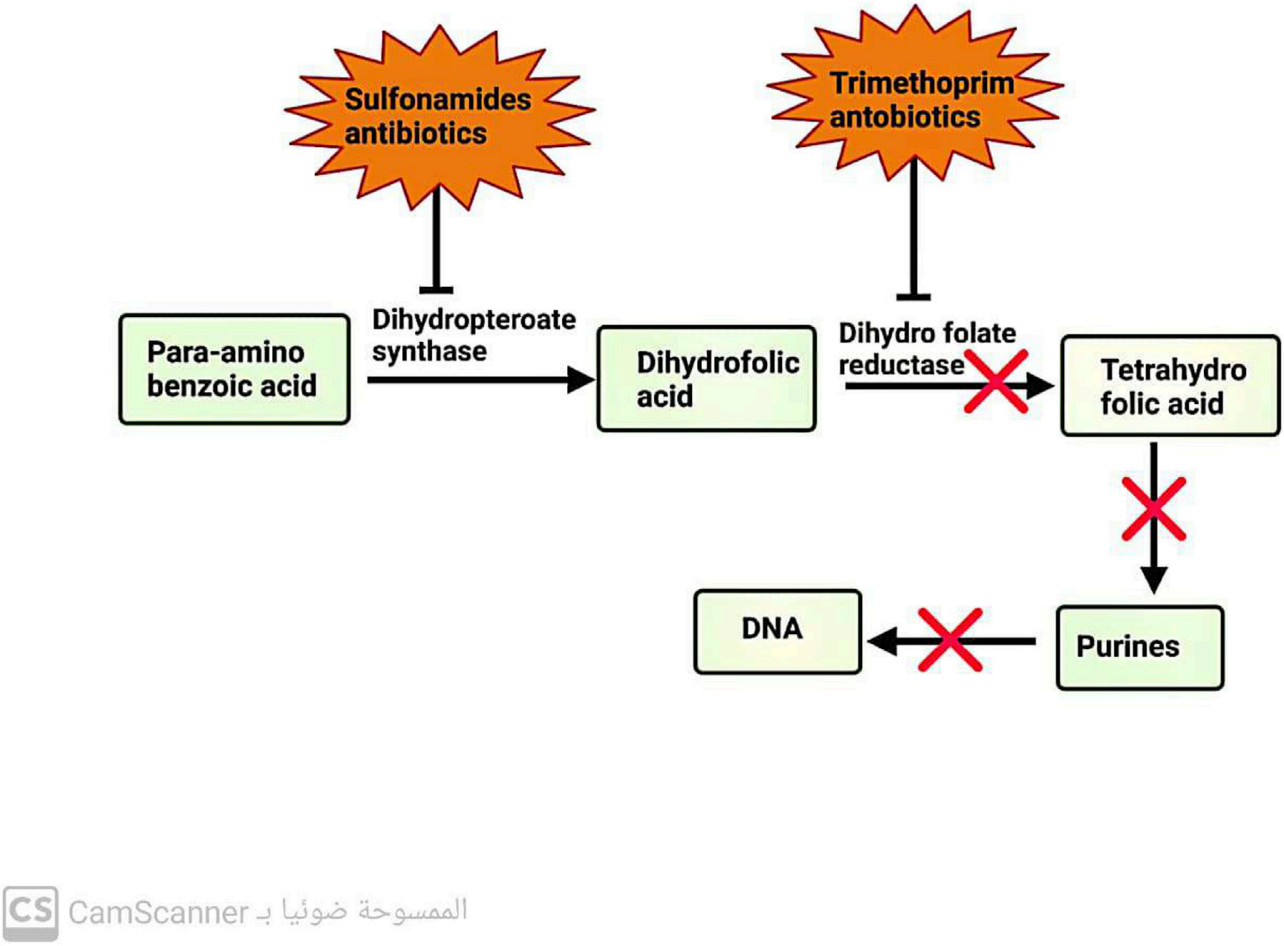
Mechanism of action of antibiotics that inhibit folic acid metabolism.

These antibiotics are designed to selectively inhibit a key enzyme involved in the pathway for folic acid metabolism. Sulfonamide antibiotics target dihydropteroate synthase, an enzyme in the metabolic pathway. On the other hand, trimethoprim antibiotics target dihydrofolate reductase, another enzyme in the same pathway (Capasso and Supuran, 2014) (Figure 4).

## 4 Emergence of antibiotic resistance

Dr. Tedros Adhanom Ghebreyesus, CEO of the World Health Organization (WHO), has warned that the global epidemic of antibiotic resistance poses a significant threat to a century of progress in healthcare and the achievement of sustainable development goals (Sarkar et al., 2021). According to current predictions, it is expected that in a quarter of a century, almost 100% of bacteria will be resistant to most antibiotics used in medicine (Kaur Sodhi and Singh, 2022; Murray et al., 2022). Experts also predict that the number of deaths due to antimicrobial resistance may rise to 10 million by the middle of the twenty-first century, up from the current figure of over 700,000 per year (Romandini et al., 2021).

In response to this alarming situation, the WHO proposed the ranking of bacteria with the highest trend in resistance in 2017. This list includes *Acinobacter baumannii*, *Pseudomonas aeruginosa, Enterobacteriaceae, Enterococcus faecium, Helicobacter pylori*, *Salmonella species, Campylobacter species, Staphylococcus aureus,* and *Nisseria gonorrhoeae*. These microbe species are highly resistant to multiple classes of antibiotic treatments, making them less susceptible to antibiotic therapy (Biondo, 2023).

## 5 Mechanisms of antibiotic resistance

Bacteria employ three primary mechanisms to counteract the effects of antibiotics (Zhou et al., 2015). These mechanisms are outlined below:

### 5.1 Bacteria prevent antibiotic accumulation in their cells

#### 5.1.1 Through limiting the entrance of drugs into bacterial cells

**FIGURE 5:**
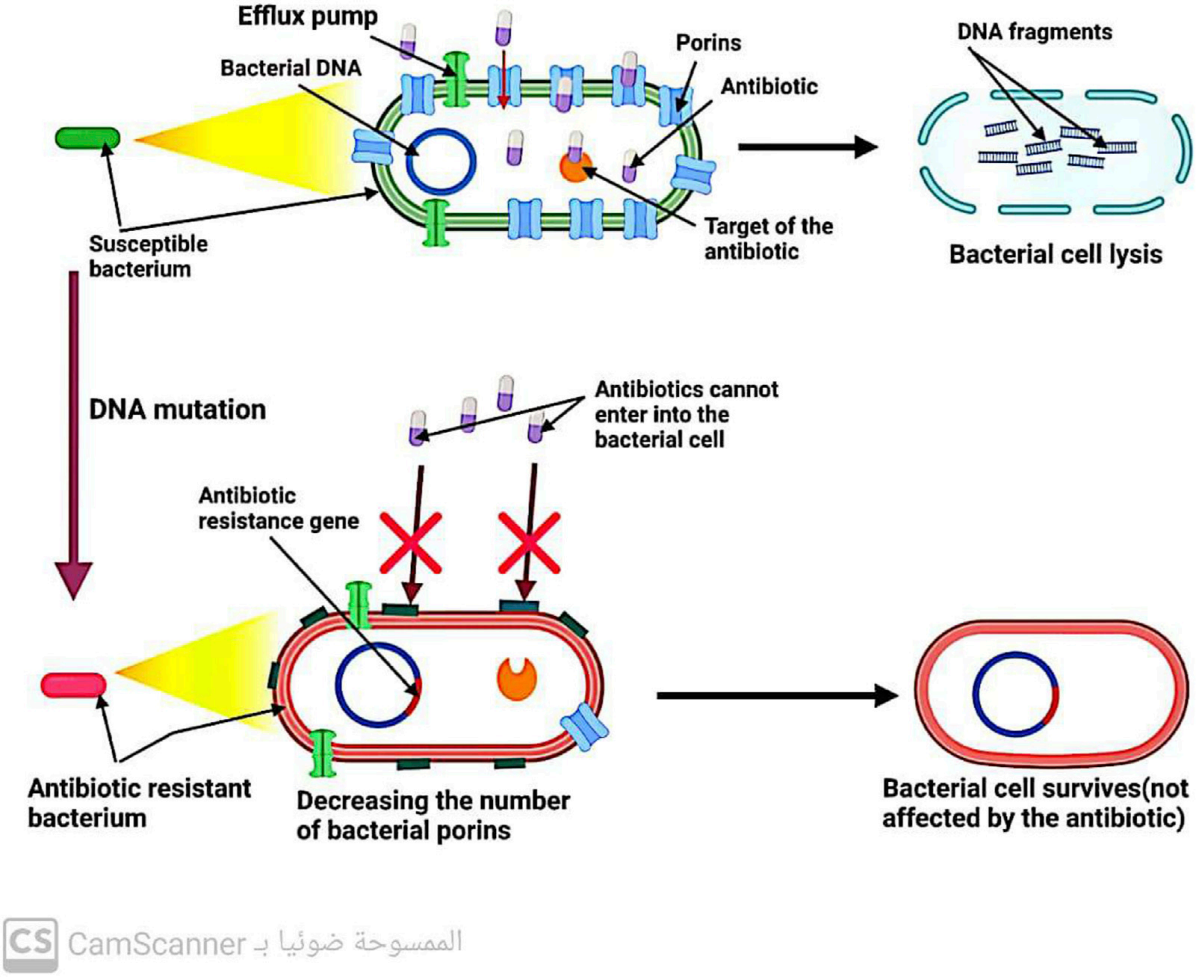
Mechanism of antibiotic resistance by decreasing antibiotic entry into the bacterial cell.

Gram-negative bacteria have porin channels in their outer membrane (Kang and Park, 2015). These channels act as gatekeepers, allowing only certain antibiotics like B-lactams and quinolones to enter the bacterial cell. Therefore, the reduced number of bacterial porins can hinder the entry of these antibiotics into the cell, leading to increased resistance to these drugs (Darby et al., 2023) (Figure 5).

#### 5.1.2 Increasing the rate at which antibiotics leave bacterial cells

**FIGURE 6:**
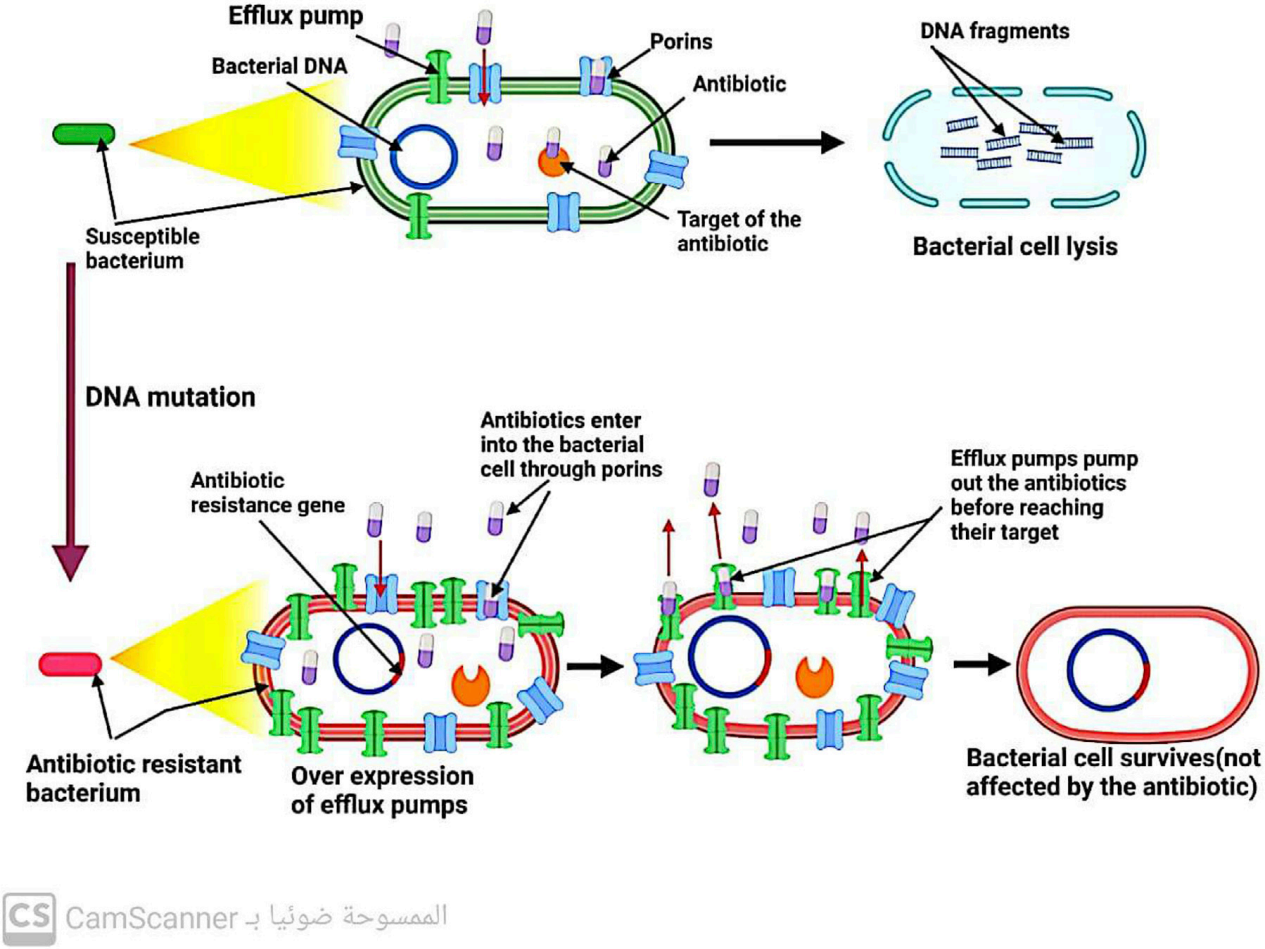
Mechanism of antibiotic resistance by increasing antibiotic exit from the bacterial cell.

Efflux pumps, located in the cytoplasmic membrane of bacteria, play a crucial role in maintaining the balance of solutes within bacterial cells. However, these pumps also contribute to antibiotic resistance by removing drugs from bacterial cells before they can reach their intended targets (Džidić, Šušković, and Kos, 2008) (Figure 6). Notably, efflux systems have been found to confer resistance (Kaur Sodhi and Singh, 2022; Shree et al., 2023; Sodhi et al., 2023). to all antibiotic classes except for polymyxin (Fernández-Billón et al., 2023). Increasing our understanding of the mechanisms underlying efflux systems may provide new strategies for combating antibiotic resistance.

### 5.2 Bacteria modify the target molecule of antibiotics

Antibiotics are designed to target specific molecules, but even the slightest alteration can prevent their binding, leading to the emergence of antibiotic resistance (Kang and Park, 2015; Shree et al., 2023).

#### 5.2.1 Modifications to the ribosomal 30s or 50s subunits

**FIGURE 7:**
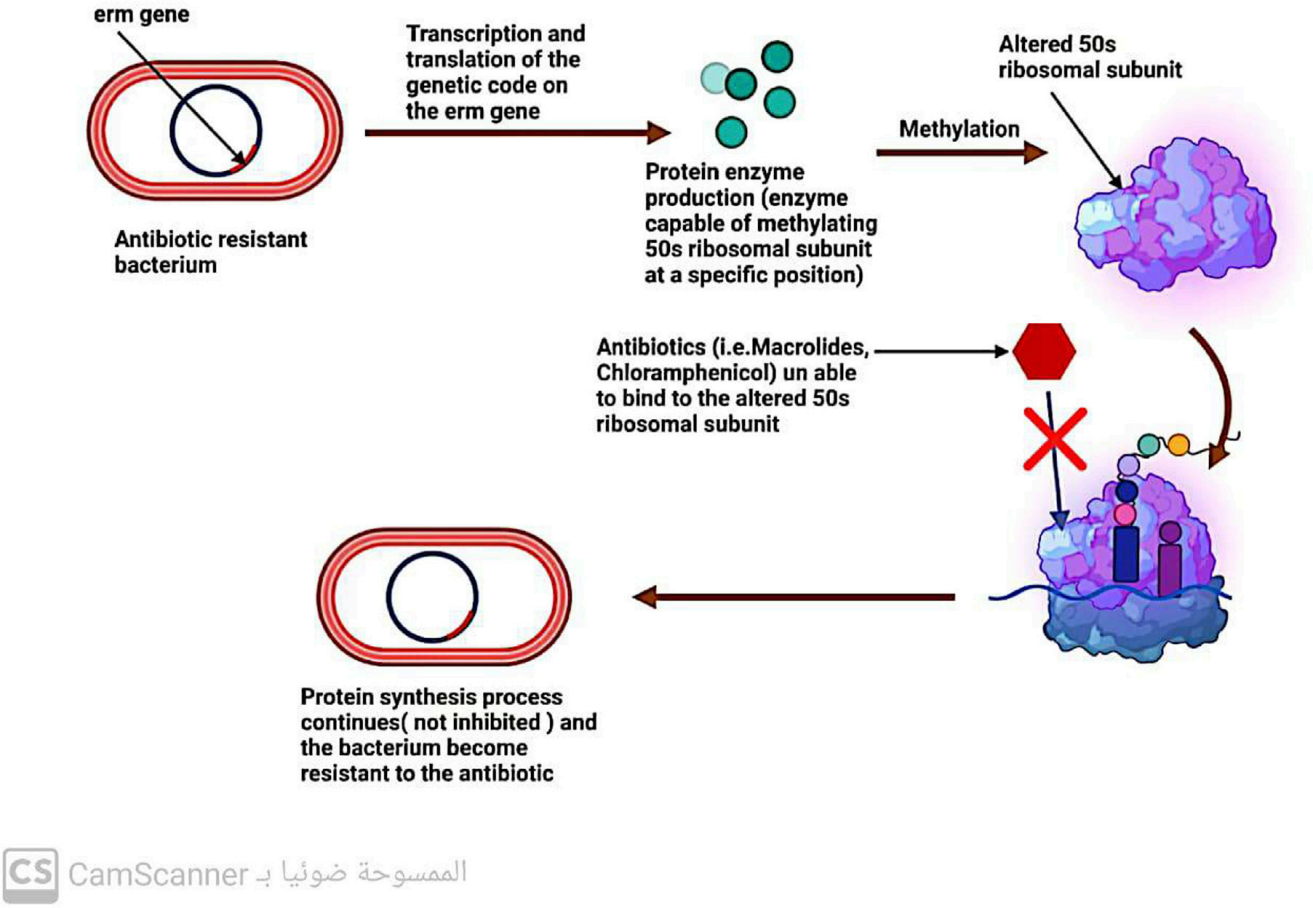
Mechanism of antibiotic resistance by alteration in 50 s ribosomal subunit.

One-way bacteria can develop resistance to drugs that affect protein production is by modifying their ribosomal 30S or 50S subunits (Kaur Sodhi and Singh, 2022; Sodhi et al., 2023) (Figure 7). This type of resistance is observed with antibiotics such as aminoglycosides, tetracycline, macrolides, chloramphenicol, lincosamides, and streptogramin (Tenover, 2006; Fernández-Billón et al., 2023).

#### 5.2.2 Changes in penicillin-binding protein (PBP)

**FIGURE 8:**
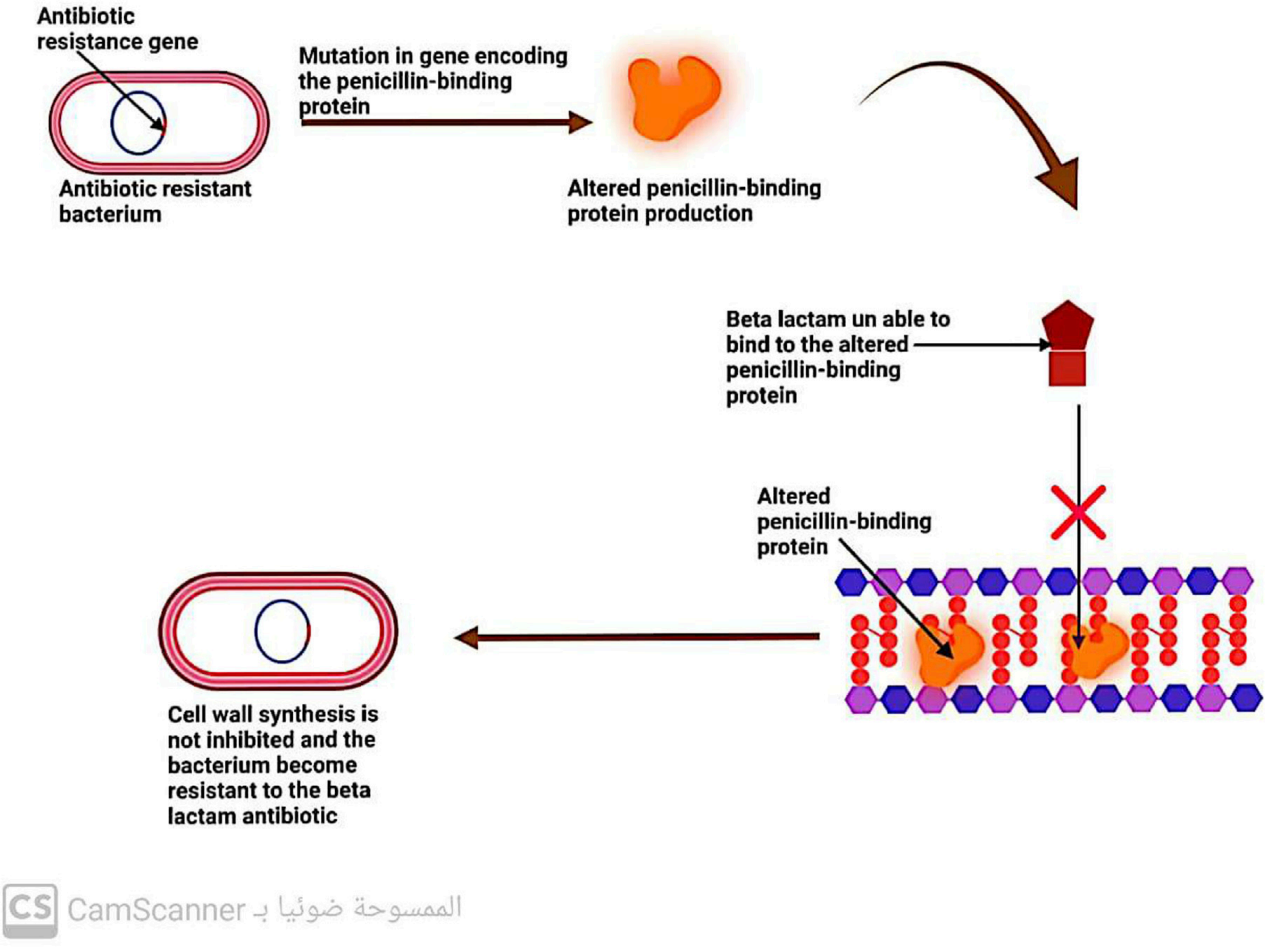
Mechanism of antibiotic resistance by alteration in penicillin-binding protein.

Penicillin-binding proteins (PBPs) are enzymes known as transpeptidases, which play a vital role in cross-linking peptidoglycan precursors during the biosynthesis of bacterial cell walls. As these enzymes are the primary targets of β-lactam antibiotics, any changes in their structure or function can lead to bacterial resistance to these drugs (Kaur Sodhi and Singh, 2022; Sodhi et al., 2023) (Figure 8).

#### 5.2.3 Changes in DNA gyrase and topoisomerase enzymes

**FIGURE 9:**
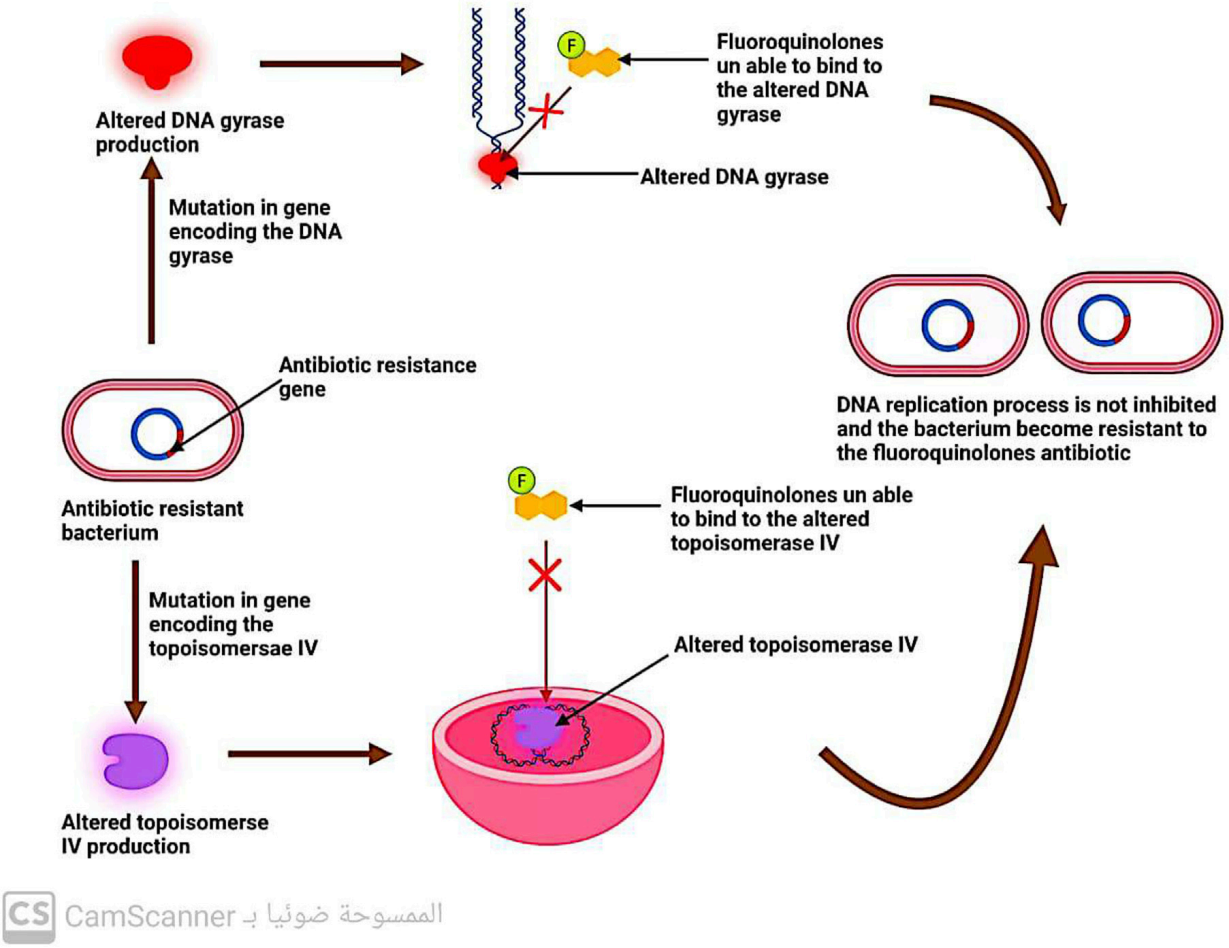
Mechanism of antibiotic resistance by alteration in DNA gyrase and topoisomerase IV.

DNA replication involves the enzymes DNA gyrase and topoisomerase (Hirsch and Klostermeier, 2021). Quinolone antibiotics specifically target these two enzymes, which is why modifications in their structure can lead to bacterial resistance against quinolones (Fàbrega et al., 2009) (Figure 9).

#### 5.2.4 Changes in D-alanyl-D-alanine

The peptidoglycan precursors contain a dipeptide residue known as D-alanyl-D-alanine, which plays a crucial role in cell wall formation (Peschel et al., 2000). Alterations to this D-alanyl-D-alanine residue can lead to bacterial resistance to antibiotics that target it (Džidić et al., 2008).

#### 5.2.5 Protection of ribosome

Tetracycline antibiotics are known to target the ribosomal 30S subunit, but the ribosome has defense mechanisms that can resist their action (Kang and Park, 2015).

#### 5.2.6 Alteration in RNA polymerase enzyme importing resistance to rifampicin antibiotics

Rifampicin is an antibiotic commonly used to treat bacterial infections. It works by inhibiting the RNA synthesis process in bacteria, specifically by binding to the beta-subunit of the DNA-dependent RNA polymerase enzyme (Hasan et al., 2021). This binding prevents the enzyme from effectively transcribing DNA into RNA, leading to the inhibition of bacterial growth and ultimately causing cell death.

However, bacteria can develop resistance to rifampicin through alterations in the RNA polymerase enzyme. Mutations in the rpoB gene, which encodes the beta-subunit of RNA polymerase, can confer resistance to rifampicin (Patel et al., 2023). These mutations can affect the binding affinity between rifampicin and the RNA polymerase enzyme, reducing the ability of the antibiotic to inhibit RNA synthesis.

The alterations in the RNA polymerase enzyme that result in rifampicin resistance can have several consequences. One of the effects is the alteration of the levels of peptidoglycan precursors, which are essential components of the bacterial cell wall. Changes in the levels of these precursors can impact the integrity and stability of the cell wall, potentially affecting the susceptibility of bacteria to other antibiotics, such as beta-lactams (Patel et al., 2023).

It is important to note that rifampicin resistance can arise through various mechanisms, and alterations in the RNA polymerase enzyme are just one of them. Other mechanisms include the acquisition of resistance genes through horizontal gene transfer and the overexpression of efflux pumps that can actively remove rifampicin from the bacterial cell (Hasan et al., 2021).

### 5.3 Bacteria inactivate the antibiotic by enzymes

**FIGURE 10:**
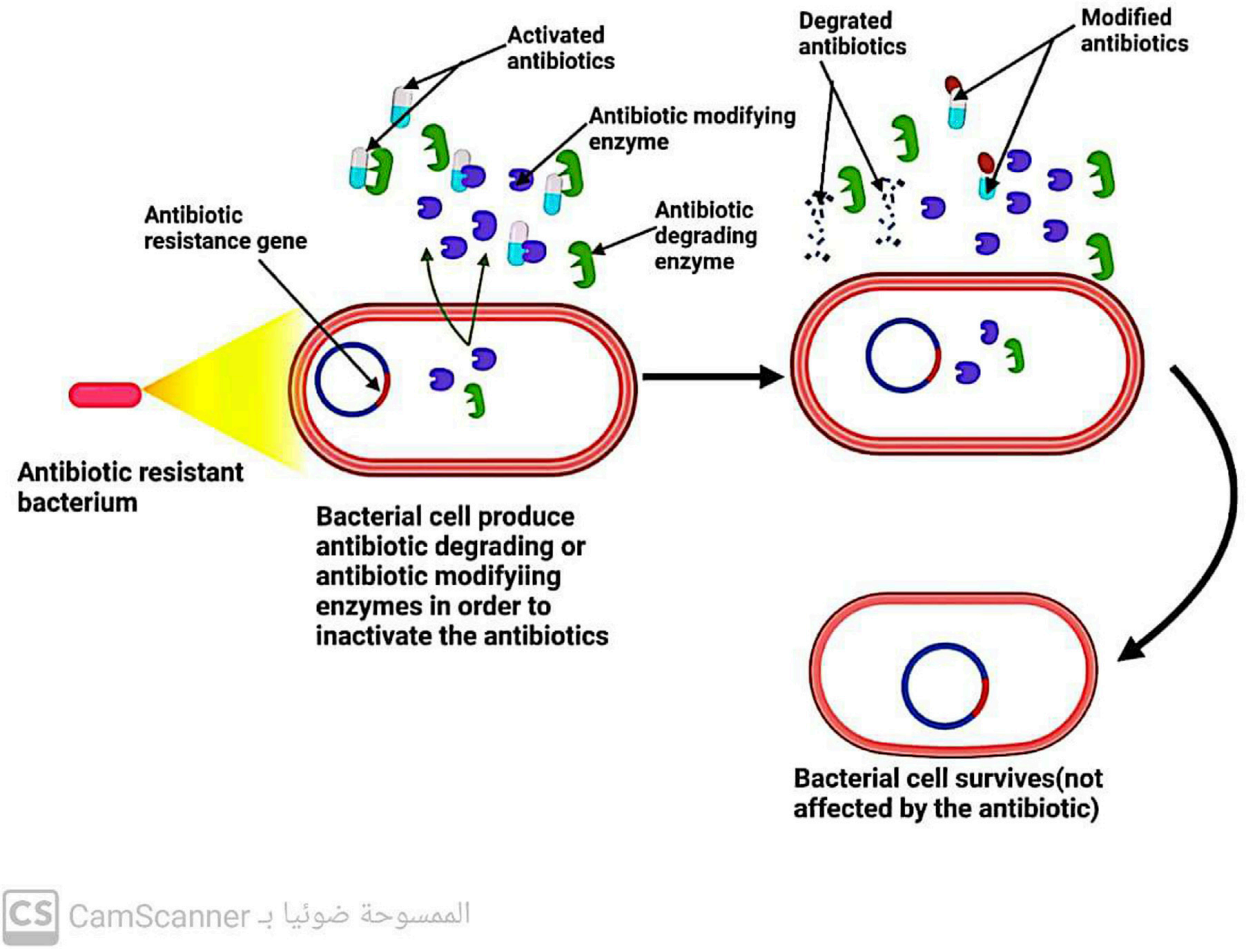
Mechanism of antibiotic resistance by inactivation of the antibiotic.

Three key enzymes are responsible for antibiotic inactivation (Figure 10). These enzymes include the following:

#### 5.3.1 Beta-lactamases enzymes

These enzymes produced by bacteria have the ability to break down all B-lactam antibiotics that are bonded with ester and amide. This leads to the development of resistance in bacteria that can produce beta-lactamase enzymes toward beta-lactam antibiotics (Fernández-Billón et al., 2023).

#### 5.3.2 Aminoglycoside modifying enzymes (AGES)

Enzymes are known to play a crucial role in antibiotic resistance. Specifically, enzymes such as aminoglycoside-modifying enzymes (AMEs) have been found to prevent the attachment of aminoglycoside antibiotics to their ribosomal target (Strateva and Yordanov 2009). These enzymes are present in various bacterial strains, including *E. faecalis*, *S. aureus*, and *S*. *pneumoniae*. In addition to their role in preventing antibiotic attachment, these enzymes also aid in conferring resistance to aminoglycosides and fluoroquinolones (Kang and Park, 2015). Thus, the presence of AMEs in bacterial strains is a major concern in the field of antibiotic resistance, as it poses a significant challenge to the effectiveness of these antibiotics in treating bacterial infections.

#### 5.3.3 Chloramphenicol-acetyl-transferases enzymes

Enzymes known as chloramphenicol-acetyltransferases modify the antibiotic chloramphenicol by acetylating its hydroxyl group, resulting in an altered form of the antibiotic that is unable to bind to its ribosomal target. Consequently, bacteria possessing the chloramphenicol-acetyltransferase enzyme are resistant to chloramphenicol antibiotics, rendering them ineffective (Varela et al., 2021).

## 6 Spread of antibiotic resistance among bacteria

**FIGURE 11:**
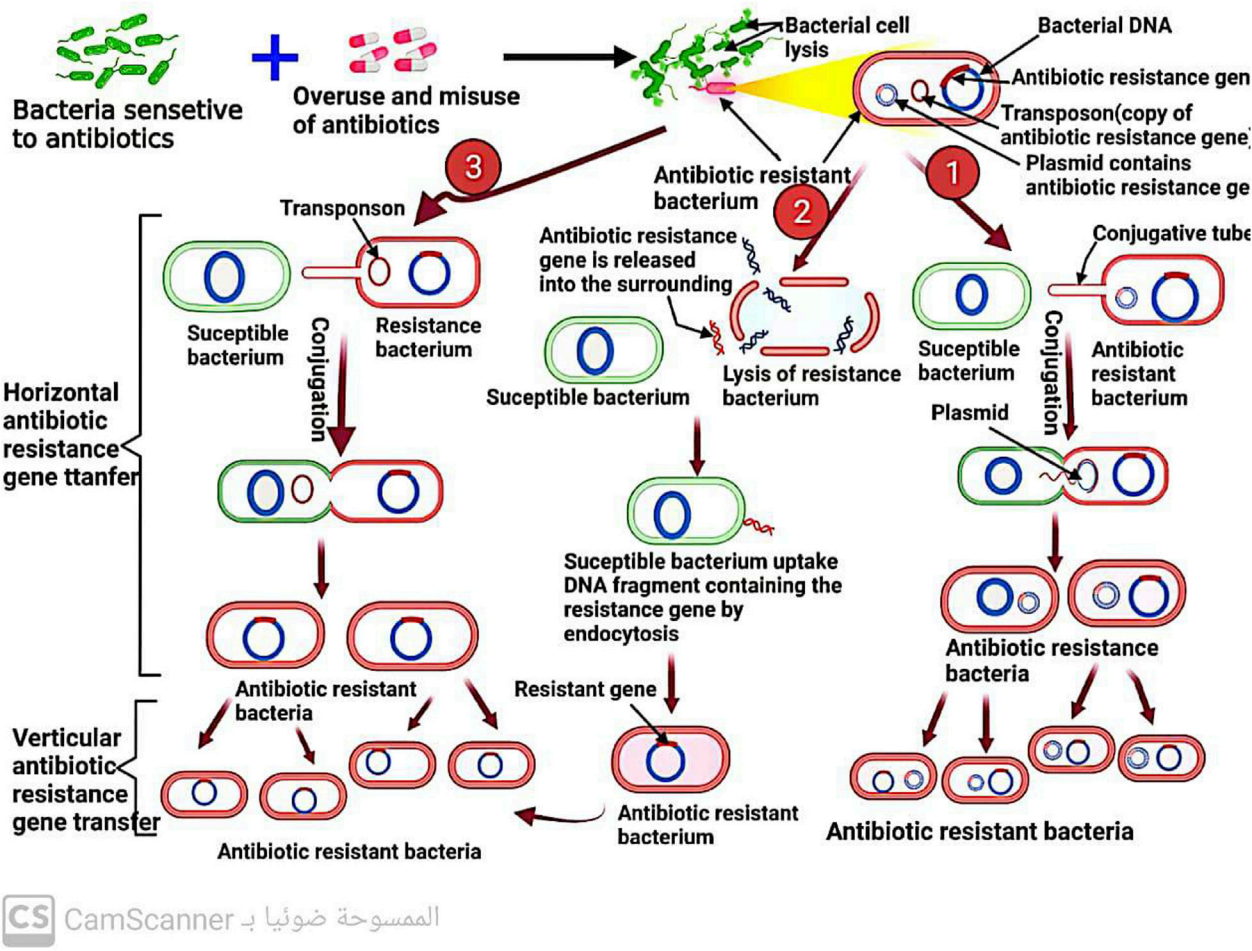
Mechanism of antibiotic resistance spread among bacteria by conjugation and transformation.

When a microorganism is able to survive or grow in an antibiotic concentration that would normally inhibit or kill other organisms of the same species, it is considered resistant (Kester and Fortune, 2014; Kaur Sodhi and Singh, 2022). In clinical practice, the terms “susceptible” and “resistant” are often used to describe the likelihood of successful treatment with antibiotics (Sabtu, Enoch, and Brown, 2015). Resistance is more likely to occur when a patient is unable to attain the concentration of antibiotic necessary to inhibit or kill the bacteria (Ordway et al., 2003). Microorganisms can either inherently possess resistance to an antibiotic or acquire it after exposure (Premlatha, 2019). The development of resistance can occur through gene mutations or direct transfer of resistance genes (Sodhi et al., 2023), which can be carried on plasmids (mobile genetic elements) (Kaur Sodhi and Singh, 2022) and transmitted through conjugation (Shree et al., 2023), or through the direct transfer of naked DNA through transformation (Shree et al., 2023) (Figure 11) or the transfer of similar DNA through bacteriophages (Kaur Sodhi and Singh, 2022; Shree et al., 2023), a process known as transduction. Even among bacteria of different species, genetic material, including antibiotic-resistance genes, can spread rapidly (Džidić et al., 2008). It was reported that heavy metals (Sodhi et al., 2023) and biofilm formation (Shree et al., 2023) increase the spread of antibiotic resistance among bacteria.

Different resistant bacteria can travel through various means, allowing them to spread and potentially cause infections in different settings. While the specific mechanisms of travel may vary depending on the bacteria and the environment, there are several common routes through which resistant bacteria can spread. For example:

1. Person-to-person transmission: Resistant bacteria can be transmitted directly from one person to another through close contact. This can occur through physical contact, such as touching or shaking hands with an infected person, or through respiratory droplets when an infected person coughs or sneezes (Ohmagari, 2014).
2. Contaminated surfaces: Resistant bacteria can survive on surfaces for extended periods. When a person touches a contaminated surface, such as doorknobs, countertops, or medical equipment, they can transfer the bacteria to their hands. If they then touch their face or mouth, the bacteria can enter their body and potentially cause an infection (Ohmagari, 2014).
3. Healthcare settings: Hospitals and healthcare facilities can be hotspots for the spread of resistant bacteria. Factors such as poor hygiene practices, inadequate infection control measures, and the close proximity of patients can contribute to the transmission of bacteria. Healthcare workers can inadvertently spread resistant bacteria from one patient to another if proper hand hygiene and infection control protocols are not followed (Clements et al., 2008; Tacconelli et al., 2014).
4. Animal-to-human transmission: Resistant bacteria can also be transmitted from animals to humans. This can occur through direct contact with infected animals or through the consumption of contaminated food products, such as meat or dairy products. Farm animals, in particular, can harbor resistant bacteria due to the use of antibiotics in agriculture (Garcia-Graells et al., 2012).
5. Travel and international spread: Resistant bacteria can be carried across borders through international travel. People who are infected or colonized with resistant bacteria can unknowingly spread them to other countries. This can contribute to the global dissemination of resistant strains and make it more challenging to control their spread (Ohmagari, 2014).

It is important to note that the specific mechanisms of travel and transmission can vary depending on the bacteria and the setting. Additionally, the spread of resistant bacteria can be influenced by factors such as poor hygiene, inadequate sanitation, and suboptimal infection control practices (Tacconelli et al., 2014).

## 7 Factors affecting the resistance of antibiotics

The emergence of antibiotic resistance is accelerated by the under, over, or improper use of antibiotics (Anwar, Iqbal, and Saleem, 2020; Kaur Sodhi and Singh, 2022; Shree et al., 2023; Sodhi et al., 2023). The indiscriminate use of antibiotics, which promotes antibiotic resistance, is caused by a variety of factors, including patients’ noncompliance with prescribed treatment and demand, irrational use of antibiotics by prescribers in human medicine, drug advertising, dispensing doctors, antibiotic use in agriculture, poor antibiotic quality, inadequate surveillance, and susceptibility testing. Despite their knowledge of a patient’s diagnosis, doctors and prescribers can be heavily influenced by patient demand, which can contribute to antimicrobial and antibiotic resistance (Acharya and Wilson, 2019).

Patients may discontinue their treatment once they begin to feel better, forget to take their prescriptions, or only purchase a portion of the medication. In such cases, increased physician-patient interaction is often necessary to ensure proper compliance with treatment (Carlet et al., 2012). Additionally, antibiotics are readily available in pharmacies without a prescription, which further contributes to their misuse by patients (Darj, Newaz, and Zaman, 2019; Machowska and Lundborg, 2019).

The pharmaceutical industry also plays a role in promoting antibiotic misuse through advertising. For example, some advertisements claim that certain antibiotics, such as Ciprofloxacin, are the best option for at-risk patients. In the past, advertisements in the Philippines encouraged the use of lincomycin for pharyngitis/tonsillitis and clindamycin for upper respiratory tract infections, despite the fact that these conditions are often caused by viral infections that do not require antibiotics (Ladd, 2005; Tanday, 2016). Overall, inappropriate antibiotic use is a multifaceted issue that requires cooperation between healthcare providers, patients, and the pharmaceutical industry to address.

Medical professionals have a significant impact on the development of antibiotic resistance in bacteria. It is common for doctors to prescribe broad-spectrum antibiotics when narrow-spectrum ones would be more appropriate (Mincey and Parkulo, 2001; Om et al., 2016). The prescribing patterns of doctors can vary, and studies have shown that 30%–60% of patients receive more antibiotics than necessary. Additionally, incorrect prescriptions and recommendations from untrained medical professionals pose a significant risk. A related study found that private practitioners often prescribe unnecessary medications (Thakolkaran et al., 2017).

Hospitals and clinics are significant contributors to the development of microbial resistance to antibiotics (Shree et al., 2023). Inadequate infection control protocols, such as failure to wash hands or change gloves regularly, have been identified as factors contributing to this problem (Weinstein, 2001). Another issue is the use of poor-quality antibiotics. This problem persists due to the lack of quality compliance and monitoring, leading to the use of expired and counterfeit antibiotics (Ayukekbong, Ntemgwa, and Atabe, 2017). Inappropriate use of antibiotics in animals is also a concern. Some antibiotics are administered to animals to boost their growth and prevent sickness (Manishimwe, Nishimwe, and Ojok, 2017). However, the surveillance and susceptibility testing of antibiotics are insufficient (Tebano et al., 2020) (Table 2).

**TABLE 2:** Factors causing antibiotic resistance in different sectors and some proposed solutions to overcome these factors.

Sector	Causes of antibiotic resistance	Proposed solutions
**Human Health**	➢ Prescription and use of antibiotics for viral infections, including Influenza, upper respiratory tract infections, pharyngitis, tonsillitis, and the common cold (Ladd, 2005; Tanday, 2016).	➢ Physicians should not prescribe antibiotics for viral infections.
	➢ Patients are not taking the entire course of antibiotics and interrupting their treatment when they feel better (Carlet et al., 2012).	➢ Patients should take their entire course of antibiotics even if they feel better.
	➢ Self-medication and misuse of antibiotics by patients because it is readily available in the pharmacy, and they can buy them without having a prescription (Darj et al., 2019; Machowska and Cecilia Stålsby, 2019).	➢ Banning the sale of antibiotics in pharmacies without a prescription.
	➢ Overprescription of un- necessary antibiotics, especially broad-spectrum antibiotics, by physicians (Mincey and Parkulo, 2001; Om et al., 2016).	➢ Physicians should only prescribe the necessary antibiotics for the disease without prescribing the unnecessary ones.
	➢ Poor infection control practices in hospitals and clinics, like hand washing and changing gloves (Weinstein, 2001).	➢ Attention should be paid to developing infection control practices in hospitals and clinics.
	➢ Lack of quality compliance and monitoring in hospitals and clinics results in expired and counterfeit antibiotics being used (Ayukekbong et al., 2017).	➢ Attention should be paid to quality compliance in hospitals and clinics to prevent the use of expired and counterfeit antibiotics.
**Animal husbandry**	➢ Overusing antibiotics in animal feeds for growth and disease control (Anwar et al., 2020; Kaur Sodhi and Singh, 2022; Shree et al., 2023; Sodhi et al., 2023).	➢ Establishing guidelines for prudent usage.
**Agriculture**	➢ Overusing antibiotics as pesticides for disease control in plants (Anwar et al., 2020; Kaur Sodhi and Singh, 2022; Shree et al., 2023; Sodhi et al., 2023).	➢ Establishing guidelines for prudent usage.
**Aquaculture**	➢ Overusing antibiotics in fish farming (Anwar et al., 2020; Kaur Sodhi and Singh, 2022; Shree et al., 2023; Sodhi et al., 2023).	➢ Establishing guidelines for prudent usage.

## 8 Alternative approaches to combat antibiotic resistance

We are now in grave danger because of the global antibiotic resistance crisis, which is constantly increasing. Therefore, we should focus on finding solutions to fight this resistance crisis. There are several approaches to fight this crisis which we discuss in this review article as follows:

### 8.1 Discovery of new antibiotics

In 2015, researchers discovered a new antibiotic called teixobactin, which demonstrated bactericidal activity against *S. aureus*, *Clostridium difficile*, and *Bacillus anthracis* (Piddock, 2015). On 20 February 2020, researchers published an article in Cell titled “A Deep Learning Approach to Antibiotic Discovery.” Using artificial intelligence, they discovered a new antibiotic called halicin, which showed bactericidal activity against a broad spectrum of pathogenic and resistant bacteria (Stokes et al., 2020).

On 26 September 2022, researchers published an article in Nature Microbiology titled “Computational identification of a systemic antibiotic for Gram-negative Bacteria.” Using computational screening, they discovered a new antibiotic called dynobactin, which demonstrated potent bactericidal activity against dangerous Gram-negative bacteria resistant to other antibiotics (Miller et al., 2022).

The dysfunctional R&D market for detecting new antibiotics refers to the challenges and limitations faced in the research and development of new antibiotics. These challenges include economic, regulatory, and scientific barriers that hinder the discovery and development of effective antibiotics to combat bacterial infections. Some of the key issues in the dysfunctional R&D market for detecting new antibiotics include:

#### 8.1.1 A-economic challenges

*Limited financial incentives:* The high cost and low profitability of developing new antibiotics have led to a lack of investment from pharmaceutical companies (Clardy, Fischbach, and Walsh, 2006).

*Long development timelines:* The lengthy and expensive process of developing new antibiotics makes it less attractive for companies to invest in this area (Clardy et al., 2006).

#### 8.1.2 B-regulatory challenges

*Stringent regulatory requirements:* The regulatory approval process for new antibiotics is complex and time-consuming, leading to delays in bringing new drugs to market (McDevitt and Rosenberg, 2001).

*Limited guidance on clinical trial design:* There is a lack of clear guidelines for conducting clinical trials for antibiotics, which can further hinder the development process (McDevitt and Rosenberg, 2001).

#### 8.1.3 C-scientific challenges

*Antibiotic resistance:* The rise of antibiotic-resistant bacteria poses a significant challenge in the development of new antibiotics (McDevitt and Rosenberg, 2001).

*Limited understanding of bacterial biology:* Despite advances in genomics and other technologies, there is still much to learn about the biology of bacteria and their mechanisms of resistance (McDevitt and Rosenberg, 2001).

### 8.2 Antibiotic adjuvants

Antibiotic adjuvants are compounds that do not directly kill bacteria but instead enhance an antibiotic’s effectiveness by inhibiting resistance mechanisms. For example, beta-lactamase inhibitors are small-molecule antibiotic adjuvants. Beta-lactamase inhibitors, when combined with beta-lactam antibiotics, have been used successfully for over 30 years to treat Gram-positive and Gram-negative infections. Their use has been extensively documented (Melander and Melander, 2017).

### 8.3 Nano antibiotics

Nanoscale antibiotics, which consist of pure antibiotic molecules between 1 and 100 nm in size or antibiotic molecules physically attached to nanoparticles, represent one beneficial use of nanotechnology (Soares et al., 2018; Singh and Sodhi, 2023). By reengineering antibiotics at the nanoscale, this new antimicrobial approach revives the arsenal of available medications by making them effective against various clinically important microorganisms. Unlike their bulk chemical counterparts, nanoantibiotics have different physicochemical properties and increased potency (Mamun et al., 2021). Because nanoscale drug delivery systems can transport and bind to intracellular targets, reducing bacterial growth and metabolism and ultimately causing cell death, a medication delivered via or incorporated into nanoparticles at the same dose has significantly greater inhibitory effects on bacterial growth (Jijie et al., 2017; Singh and Sodhi, 2023).

### 8.4 Botanicals

Plants produce secondary metabolites, including alkaloids, flavonoids, phenolics, quinones, tannins, coumarins, terpenes, lectins, and saponins. These secondary metabolites exhibit antimicrobial activity against various microorganisms (Gupta and Birdi, 2017).

### 8.5 Bacteriophages

According to the National Institute of Health (NIH, 2014), bacteriophages are innovative elements that could combat microbial resistance (Shree et al., 2023; NIAID’s Antibacterial Resistance Program, 2014). Numerous studies applied bacteriophages on humans and animals to treat various bacterial diseases and showed positive, effective results. These bacterial pathogens include *Shigella dysenteriae* (Chanishvili, 2012), *Vibrio cholera* (Chanishvili, 2012), *P. aeruginosa* (Watanabe et al., 2007), *C. difficile* (Meader et al., 2013), Vancomycin-resistant *E. faecium* (Biswas et al., 2002), β-lactamase-producing *E. coli* (Wang et al., 2006), imipenem resistant *P. aeruginosa* (Wang et al., 2006), *Acinetobacter baumannii* (Yuan et al., 2019), *E. coli* (Pouillot et al., 2012), MDR-*S. aureus* (Fish et al., 2016), unclassified bacterial dysentery (Chanishvili and Sharp, 2008), *S. typhi* (Kutateladze and Adamia, 2008), and antibiotic-resistant *P*. *aeruginosa* (Wright et al., 2009).

## 9 Conclusion

This review article has discussed various aspects related to antibiotics, their mechanisms of action, the problem of antibiotic resistance, and potential solutions to combat resistance. Antibiotics have diverse applications in different fields but their use remains controversial owing to resistance issues. Antibiotics act through four primary mechanisms to kill or inhibit the growth of bacteria. However, bacteria have evolved mechanisms to become resistant to antibiotics, diminishing the effectiveness of these drugs. Given the limited capabilities of traditional antibiotics due to resistance, we discussed several promising alternative approaches, including the discovery of new antibiotics, the use of antibiotic adjuvants and nanoparticle-based antibiotics, botanicals, and bacteriophages. Although developing new antibiotics is challenging, a combination of complementary strategies such as nanoantibiotics, adjuvants, botanicals, and phage therapy could help address the resistance crisis.
